# ChestX-Transcribe: a multimodal transformer for automated radiology report generation from chest x-rays

**DOI:** 10.3389/fdgth.2025.1535168

**Published:** 2025-01-21

**Authors:** Prateek Singh, Sudhakar Singh

**Affiliations:** Biomedical Engineering Department, School of Bioengineering and Biosciences, Lovely Professional University, Punjab, India

**Keywords:** medical report generation, multimodal transformers, swin transformer, DistilGPT, vision-language models, radiology workflow

## Abstract

Radiology departments are under increasing pressure to meet the demand for timely and accurate diagnostics, especially with chest x-rays, a key modality for pulmonary condition assessment. Producing comprehensive and accurate radiological reports is a time-consuming process prone to errors, particularly in high-volume clinical environments. Automated report generation plays a crucial role in alleviating radiologists' workload, improving diagnostic accuracy, and ensuring consistency. This paper introduces *ChestX-Transcribe*, a multimodal transformer model that combines the Swin Transformer for extracting high-resolution visual features with DistilGPT for generating clinically relevant, semantically rich medical reports. Trained on the Indiana University Chest x-ray dataset, *ChestX-Transcribe* demonstrates state-of-the-art performance across BLEU, ROUGE, and METEOR metrics, outperforming prior models in producing clinically meaningful reports. However, the reliance on the Indiana University dataset introduces potential limitations, including selection bias, as the dataset is collected from specific hospitals within the Indiana Network for Patient Care. This may result in underrepresentation of certain demographics or conditions not prevalent in those healthcare settings, potentially skewing model predictions when applied to more diverse populations or different clinical environments. Additionally, the ethical implications of handling sensitive medical data, including patient privacy and data security, are considered. Despite these challenges, *ChestX-Transcribe* shows promising potential for enhancing real-world radiology workflows by automating the creation of medical reports, reducing diagnostic errors, and improving efficiency. The findings highlight the transformative potential of multimodal transformers in healthcare, with future work focusing on improving model generalizability and optimizing clinical integration.

## Introduction

Chest x-rays remain one of the most widely used diagnostic tools in healthcare for assessing pulmonary disorders. However, interpreting these images and generating accurate, detailed reports is a time-consuming and subjective task, particularly in high-volume clinical environments. Automating this process with deep learning holds the potential to streamline diagnostic workflows and reduce the burden on radiologists. A significant challenge in healthcare is the prevalence of diagnostic errors, with studies (1) indicating that nearly everyone will experience a diagnostic error at least once in their lifetime. Automating the report generation process can mitigate these errors, enhancing diagnostic accuracy and consistency. By relying on automated systems for report preparation, healthcare professionals can ensure more reliable interpretations of chest x-rays. Recent advancements in artificial intelligence (AI) have been driven by transformer architectures, which have revolutionized natural language processing (NLP) and computer vision. Vision transformers, such as Swin Transformer (2), excel at capturing intricate spatial patterns in medical images, while language models like GPT have shown remarkable ability in generating coherent, contextually accurate text.

However, these technologies have only recently begun to be explored in the context of automated clinical workflows, especially for medical image captioning. In this study, we introduce ChestX-Transcribe, a multimodal sequence-to-sequence transformer model that combines the strengths of DistilGPT (3) for generating precise radiology reports with the Swin Transformer (4) for extracting high-resolution visual features from chest x-rays. By seamlessly integrating both vision and language transformers, ChestX-Transcribe offers an innovative approach to automating medical report generation. This model enhances diagnostic workflows by producing reliable, contextually appropriate medical reports, reducing the cognitive load on radiologists, and supporting faster diagnosis.

Key contributions of this study include:

• **Multimodal Transformer Architecture:** ChestX-Transcribe integrates a pre-trained Swin Transformer for high-resolution visual feature extraction from chest x-rays with DistilGPT, a distilled version of GPT-2, for language generation. This combination enables the model to effectively handle both local and global dependencies in visual data while generating coherent text, offering a robust solution for medical report generation in healthcare.
• **Efficient and Scalable Model Design:** By leveraging DistilGPT, a smaller and faster variant of GPT-2, we achieve notable improvements in model efficiency without sacrificing the quality of the generated reports. The reduced computational complexity makes the model more scalable for clinical applications where real-time processing and resource efficiency are essential.
• **Dataset Utilization and Performance Evaluation:** We evaluate the model using the widely recognized Indiana University Chest x-ray dataset (5), facilitating performance comparisons with existing state-of-the-art methods. Preliminary results demonstrate a marked improvement in BLEU and ROUGE scores, highlighting ChestX-Transcribe's ability to generate clinically relevant text, indicating its potential in real-world medical settings.
• **Projection Layer for Cross-Modality Fusion:** A key innovation in this work is the introduction of a projection layer that bridges the gap between the high-dimensional visual features from the Swin Transformer and the language model, ensuring smooth integration of image embeddings into the text generation pipeline. This layer significantly enhances the model's ability to correlate image features with accurate medical descriptions, setting our approach apart from existing methods.

This work contributes novel insights by combining advanced visual and language transformers in a cohesive model for automated medical report generation, showing the potential to improve both the efficiency and accuracy of radiology workflows.

## Literature review

Automated Radiological Report Generation is one of the techniques used to characterize the clinical aspects of chest x-ray images. It is a powerfully influential field that combines natural language processing with computer vision. Earlier approaches to report writing included retrieval of descriptions, filling of templates, and manually developed NLP techniques. Later on, automated medical report creation saw several developments, but the fundamental idea behind all of them was to use an image encoder to transform CXR images into a latent space, which was then used by a decoder to produce medical reports. The issue was classified as an image-to-sequence issue in general. The reviewed literature encompasses a wide spectrum of methodologies utilized in Automated Radiological Report Generation. This includes CNN-based models, attention-driven mechanisms, and hybrid approaches combining reinforcement learning and encoder-decoder architectures. Such diversity highlights the progression and innovation in this domain, reflecting current research trends and addressing complex challenges effectively. The idea was introduced by Allaouzi et al. (6), of using a CNN-RNN architecture to automatically produce medical reports from images. As research in the field progressed, the attention layer (2) was added in several tests, and models like (7) began fusing the standard CNN-RNN architecture with the attention mechanism to project multi-view visual features which is based on a sentence-level in a late fusion fashion. A dynamic graph paired with contrastive learning in transformers was proposed by Li et al. (8). This enhanced textual and visual representation in the work of creating medical reports. Jing et al. (9) presented a technique that combines multi-task learning and co-attention mechanism to identify aberrant patches in medical images. The author then overcame the challenge of creating long paragraph-level reports by using an LSTM-based hierarchical decoder to generate comprehensive clinical imaging reports with visual attention and labels. In the proposed model for automatic report generation from chest x-ray images, Hou et al. (10) designed an architecture consisting of three key components: an encoder, decoder, and reward module. Two branches are featured by an encoder: a CNN that extracts visual features from the input images and a multi-label classification (MLC) branch that predicts common medical notions and findings. These predictions are embedded as vectors and passed to the decoder. The decoder is constructed employing a multi-level attention hierarchical LSTM, and generates reports in two stages—first, a sentence LSTM produces topic vectors to outline the content of each sentence, followed by a word LSTM that generates the specific words for each sentence based on the topic vectors. To enhance the report quality, a reward module with two discriminators provides feedback by evaluating the generated report's quality, and this feedback is employed to train the generator using reinforcement learning. The reward and the decoder module are trained adversarial in alternating iterations. An iterative decoder with visual attention was developed by Xue et al. (11) to ensure the coherence between texts. Jianbo et al. (12) utilized the multi-view information of the IU-Xray dataset by using a Resnet152 model trained on the Chexpert dataset (13) to extract the visual features and tags' prediction from the patient's front and side images. Hierarchical LSTMs were then used to generate the report. A transformer-based neural machine translation model was put forth by Lovelace and Mortazavi (14) that made use of a fine-tuning technique to extract clinical data from the reports produced and enhance clinical consistency. To create reports from the IU-Xray dataset, a customized transformer, and an additional relational memory unit were also utilized by Chen et al. (15). A visual extractor uses trained models like VGG and Resnet to extract a set of visual attributes from the front and side chest images. These features are then sent to an encoder and decoder to produce reports. A framework for generating medical reports from chest x-rays was presented by Pino et al. (16). It primarily relies on a Template-based Report Generation (CNN-TRG) model. This model states abnormalities found in the x-ray images using preset templates and fixed words. By using templates, CNN-TRG takes a more straightforward and systematic approach than many other deep learning-based Natural Language Generation (NLG) techniques, which makes it easier to guarantee clinical accuracy in the produced reports. Variational Topic Inference (VTI), which is a novel method for automating the creation of medical image reports—a crucial task in clinical practice—was presented by Najdenkoska et al. (17). VTI successfully handles the issue of different report formats written by radiologists with differing degrees of expertise. This method makes use of conditional variational inference and deep learning techniques, with a primary emphasis on latent topics that inform sentence construction. These latent subjects help to align the visual and verbal modalities into a single latent area. The visual prior net, which encodes local visual signals from input images, the language posterior net, which records the associations between word embeddings in the generated sentences, and the sentence generator net are the three key components of VTI. Akbar et al. (18) used DenseNet121 to extract image features and in the training phase applied regularization using a dropout of 20%. For medical report generation, they used the default embedding layer of Keras, they gave both the image vector and text embedding layer for training. Lee et al. (19) presented a model that included a Cross Encoder-Decoder Transformer and a Global-Local Visual Extractor (GLVE and CEDT).The language for global characteristics such as organ size and bone shape was written using the GLVE. They employed multi-level encoding features using CEDT. Chen et al. (20) have improved the generation process through the integration of cross-modal memory networks, allowing interactions between text and visuals, among other modalities. Han et al. (21) provides a framework for combining reinforcement learning (RL) with diffusion probabilistic models to generate chest x-rays (CXRs) conditioned on diagnostic reports. Using Reinforcement Learning with Comparative Feedback (RLCF), the model refines image generation through comparative rewards, ensuring accurate posture alignment, diagnostic detail, and report-image consistency. Additionally, learnable adaptive condition embeddings (ACE) enhance the generator's ability to capture subtle medical features, leading to pathologically realistic CXRs. Parres et al. (22) introduced a two-stage vision encoder-decoder (VED) architecture for radiology report generation (RRG), combining negative log-likelihood (NLL) training and reinforcement learning (RL) optimization. Text augmentation (TA) is proposed to enhance data diversity by reorganizing phrases in reference reports, addressing data scarcity and improving report quality and variability. Pan et al. (23) proposed a chest radiology report generation method using cross-modal multi-scale feature fusion. It incorporates an auxiliary labeling module to focus on lesion regions, a channel attention network to enhance disease and location feature representation, and a cross-modal feature fusion module that aligns multi-scale visual features with textual features through memory matrices for fine-grained integration.

Also Iqra et al. (24) introduced a Conditional Self-Attention Memory-Driven Transformer model for radiology report generation. The process involved two phases: first, a ResNet152 v2-based multi-label classification model was used for feature extraction and multi-disease diagnosis. Next, the Conditional Self-Attention Memory-Driven Transformer acts as a decoder, leveraging memory-driven self-attention mechanisms to generate textual reports. Sharma et al. (25) introduced the MAIRA-Seg framework, a segmentation-aware multimodal large language model (MLLM) for radiology report generation. They trained expert segmentation models to obtain pseudolabels for radiology-specific structures in chest x-rays (CXRs). Building upon the MAIRA architecture, they integrated a trainable segmentation tokens extractor that leverages these segmentation masks and employed mask-aware prompting to generate radiology reports.

Tanno et al. (26) developed the *Flamingo-CXR* system for automated chest radiograph report generation, which was evaluated by board-certified radiologists. Their study found that AI-generated reports were deemed preferable or equivalent to clinician reports in 56.1% of intensive care unit cases and 77.7% for in/outpatient x-rays. Despite errors in both AI and human reports, the research highlights the potential for clinician-AI collaboration to improve radiology report quality.

In the clinical environment, the integration of explainable AI (XAI) systems has become increasingly important for fostering trust and ensuring that clinicians understand the rationale behind AI-driven decisions. XAI allows for transparent reporting, providing meaningful explanations for the recommendations made by AI systems, which is crucial for enhancing clinical decision-making and improving patient care. The use of a modality-specific lexicon plays a key role in ensuring that AI-generated reports are detailed, contextually relevant, and interpretable. In the context of breast cancer diagnosis, Bastos et al. (27) developed a system that incorporates semantic annotation into medical image analysis to generate clearer, more comprehensive explanations of findings, allowing clinicians to better understand AI predictions. This approach not only enhances the transparency of AI models but also ensures they are aligned with clinicians' needs, ultimately facilitating the adoption of AI systems in real-world clinical settings. Bluethgen et al. (28) developed a domain-adaptation strategy for large vision-language models to overcome distributional shifts when generating medical images. By leveraging publicly available chest x-ray datasets and corresponding radiology reports, they adapted a latent diffusion model to generate diverse, visually plausible synthetic chest x-ray images, controlled by free-form medical text prompts. This approach offers a viable alternative to using real medical images for training and fine-tuning AI models.

## Methodology

**Figure 1:**
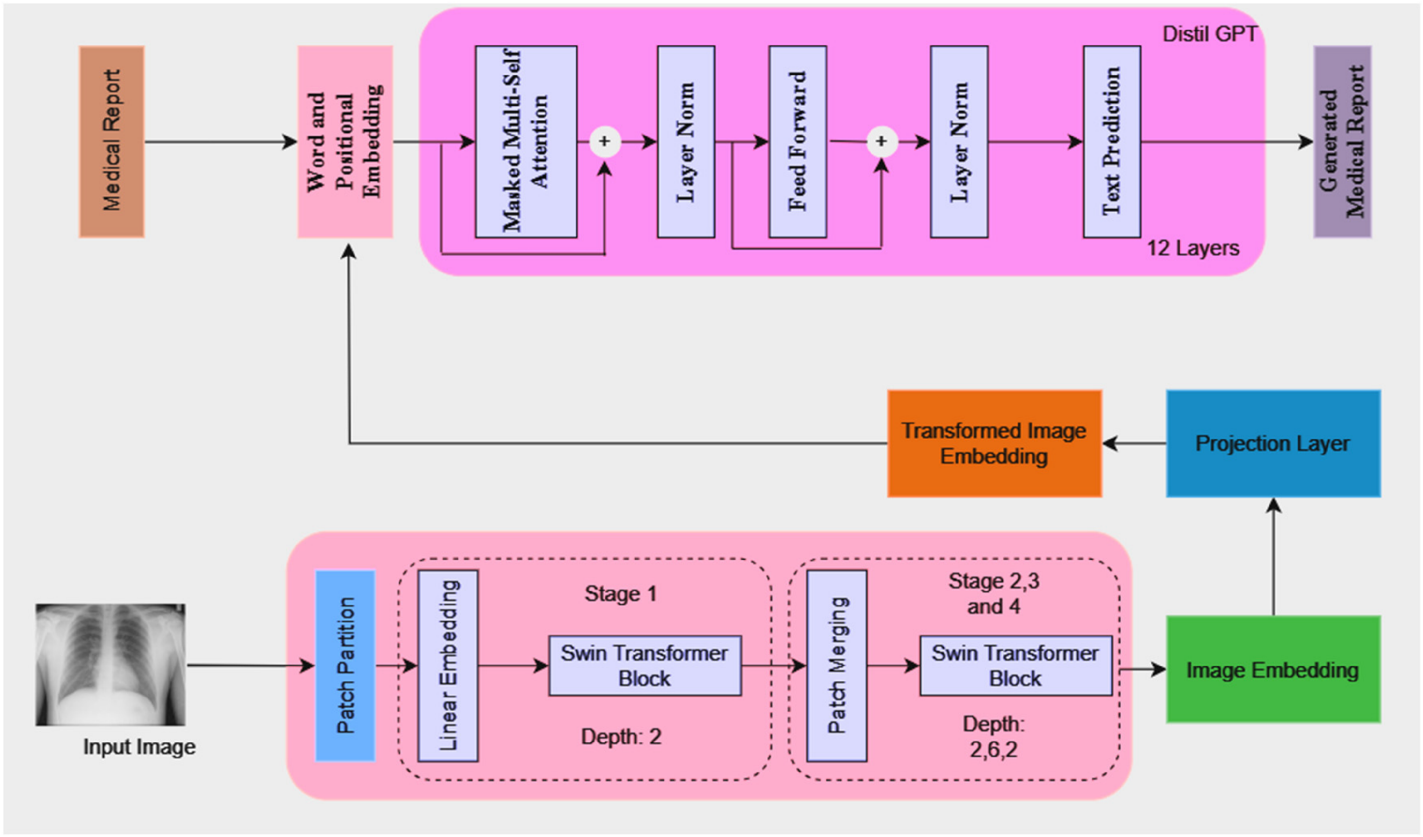
ChestX-Transcribe architecture.

The model architecture consists of a language model, a projection layer, and a vision model, as illustrated in Figure 1. Because of its architecture, the model can produce comprehensive medical reports that are shaped by the visual features that are taken out of the medical images.

### Visual model (swin transformer)

A Swin transformer model is used as the initial processing step to extract visual features from the input chest x-rays. The image is partitioned into patches and passed through the transformer blocks, which operate hierarchically to capture both local and global image information. The extracted visual features are then transformed into a high-dimensional embedding vector.

#### Working

1. Patch Partitioning and Linear Embedding: The input image *I* is split into non-overlapping patches of size M×M. The patches are then flattened and passed to a linear embedding layer. A linear embedding layer projects each flattened patch into a feature space of dimension of *C*. Each patch now becomes a “token” with a feature vector. The number of tokens *N* (Equation 1) is then calculated as:
  (1) $$N\;=\;\frac{H\;\times \;W}{M^{2}}$$
2. Window-based Multi-head Self-Attention (W-MSA): Swin transformers use window-based multi-head self-attention where self-attention is computed with non-overlapping windows. The computational complexity for global multi-head self-attention (MSA) (Equation 2) and window-based self-attention (W-MSA) (Equation 3) can be described as:
  (2) $$\Omega (MSA)\;=\;4hwc^{2}\;+\;2(hw{)}^{2}C$$
  (3) $$\Omega (W-MSA)\;=\;4hwc^{2}\;+\;2M^{2}hwC$$
  where *h* and *w* are the dimensions of the input feature map, and *M* is the window size. The first term represents the cost of computing the queries, keys, and values, while the second term captures the attention computation.
3. Shifted Window-based Multi-head Self-Attention (SW-MSA): SW-MSA is a feature introduced by Swin Transformer that enables token interactions across windows. The windows are shifted by $\frac{M}{2}$ pixels between successive layers. This enables information exchange across windows.
4. Patch Merging: After every stage, patch merging is used to downsample the resolution of the feature map while increasing the feature dimension.

### Projection layer

After extraction of the image embedding (Figure 1) from the vision model happens, a projection layer is used to align these embeddings to match the input dimension expected from the language model. This layer helps to ensure that the image representations can be effectively combined with token embeddings from the language model for joint processing. The projection layer applies a linear transformation (Equation 4) to map the image embeddings from the dimensionality $d_{image}$ to $d_{lang}$. This can be expressed as:

(4) $$z_{\;proj}\;=\;z_{image}W_{\;proj}\;+\;b_{\;proj}$$

Where:

• $W_{\;proj}\;\in R^{d_{image}\times d_{lang}}$ is the weight matrix of the projection layer.
• $b_{\;proj}\in R^{d_{lang}}$ is the bias vector.

### Language model (DistilGPT)

The language model is based on DistilGPT, which consists of 12 transformer layers. It takes token embeddings as input and predicts the next token in the sequence to generate a coherent medical report. The token embeddings are generated from the medical report text and processed through the model's transformer layers, which include masked multi-head self-attention, layer normalization, and feed-forward layers. The model also integrates the transformed image embeddings with the token embeddings at the beginning of the input sequence to condition the report generation on both the visual and textual information. The output dimension of the vision model (Swin Transformer) is set to 768 which matches the dimensionality required for the input to the language model.

We selected DistilGPT for its ability to balance performance, computational efficiency, and adaptability to domain-specific text. While larger language models (e.g., GPT-3) offer superior generalization capabilities, their computational cost makes them less feasible for clinical deployment. DistilGPT retains 97% of GPT's performance while being significantly faster and lightweight, making it ideal for generating radiology reports in real-world, high-volume settings.

### Training details

The dataset used in this study is the Indiana University Chest x-ray Dataset, which consists of 7,430 images of frontal and lateral chest x-rays belonging to 3,825 patients. Each image is paired with corresponding radiology reports that provide detailed findings regarding the patients' conditions. This dataset serves as the foundation for training the model to generate textual descriptions based on visual inputs.

#### Image preprocessing

Each x-ray image undergoes a series of transformations as included in the Swin Transformer model preprocessing pipeline. First, the image is resized to the standard dimensions which are suitable for the model's input, ensuring uniformity across all the input samples. Next, pixel values are normalized in a range between 0 and 1.

#### Text preprocessing

The textual findings from the radiology reports are tokenized using the GPT-2 tokenizer. This process involves encoding the findings into a sequence of token IDs representing the words or subwords in the text. These token IDs allow the model to interpret and process the textual information effectively. To ensure compatibility with the model's input requirements, tokenized sequences are constrained to a specified maximum length.

If a sequence exceeds this length, it is truncated to fit the required size, preventing overflow during processing. Additionally, an end-of-sequence token ID is appended to mark the conclusion of the text sequence, signaling the model when to stop generating output. In this study, the dataset consists of a total of 155,837 tokens. This token count reflects the cumulative number of tokens across all text sequences in the dataset. To maintain consistency and optimize the performance of the model, the output is restricted to a maximum of 100 newly generated tokens. This limit includes both the input prompt and the generated text. Therefore, the total length of the output is carefully controlled, ensuring that the generated sequence is neither too short nor too long, and can be evaluated consistently across experiments.

#### Training and validation loss

**Figure 2:**
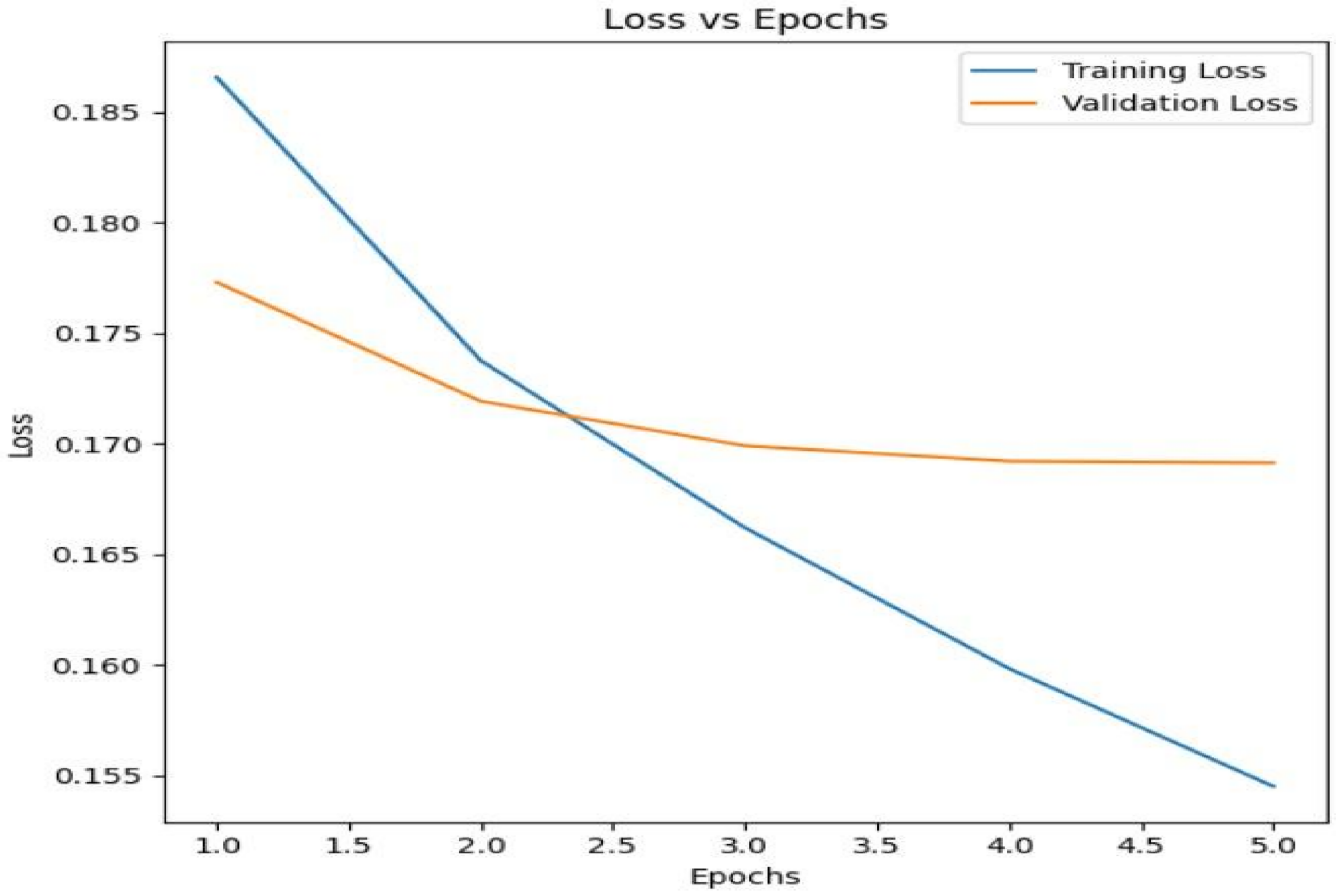
Training vs. Validation Loss (Trends Observed Over 5 Epochs).

The model was trained over 5 epochs with both training and validation losses being tracked to monitor the model's performance and prevent overfitting. Figure 2 below shows the Training Loss vs. Validation Loss over 5 epochs.

The model is learning from the data as seen by the training loss, which gradually drops throughout the epochs. Initially, the validation loss decreases along with the training loss, showing that the model is improving its generalization to unseen data. However, after approximately 2 epochs, the validation loss plateaus, suggesting that further training may lead to overfitting.

The adaptive learning rate method of the Adam optimizer, which works well for tasks involving huge datasets and parameters, was used to train the model. To maximize performance, two different learning rates were applied to various model components in this configuration. To ensure that fine-tuning happens gradually without causing significant changes, the language model's parameters were updated using a learning rate of 2e-4.

This allowed the model to preserve its pre-trained knowledge while responding to the task-specific data. On the other hand, because these layers are more task-specific and call for more substantial updates, the projection model's parameters were changed using a higher learning rate of 5e-5. By keeping a balance throughout training, this differential learning rate technique helps keep the model from underperforming or overfitting too soon.

### Evaluation metrics

1. **BLEU Score:** Bilingual Evaluation Understudy (BLEU) (29) (Equation 5) is a widely used metric in natural language processing used for evaluating the quality of text generated by the model. It measures the overlap between the generated text and the reference text by comparing n-grams (contiguous sequences of words). A higher BLEU score indicates better alignment with the ground truth captions, demonstrating the model's ability to generate coherent and relevant descriptions.
  (5) $$BLEU\;=\;BP.exp\left(\overset{N}{\underset{n=1}{\sum }}\;w_{n}\;logp_{n}\right)$$

Where;

• *BP* is the brevity penalty, which penalizes shorter sentences to encourage longer, more complete outputs.
• *p_n_* is the precision for n-grams of order n.
• *w_n_* is the weight assigned to the precision of each n-gram level.
• *N* is the maximum length of the n-grams.
2. **ROUGE-L:** The ROUGE-L (Recall-Oriented Understudy for Gisting Evaluation - Longest Common Subsequence) (30) metric measures the Longest Common Subsequence (LCS) between a generated text and a reference text. It focuses on capturing the sequence similarity while maintaining word order. ROUGE-L computes precision (Equation 6), recall (Equation 7), and F1-score based on the length of the LCS between the generated sequence *C* and the reference sequence *R*.
  $$\begin{array}{rl} LCS(C,R)= & \;\mathrm{Length}\;\mathrm{of}\;\mathrm{the}\;\mathrm{Longest}\;\mathrm{Common}\;\mathrm{Subsequence}\\ \mathrm{between}\;C\;\mathrm{and}\;R \end{array}$$
  (6) $$P\;=\;\frac{LCS(C,R)}{\left|C\right|}$$
  (7) $$R\;=\;\frac{LCS(C,R)}{\left|R\right|}$$
• *P* is the Precision.
• *R* is the Recall.
• |C| is the length of the generated text.
• |R| is the length of the reference text.
3. **ROUGE-1:** The ROUGE-1 metric evaluates the unigram overlap between the generated text and the reference text. It captures the presence of individual words from the reference in the generated sequence, measuring precision, recall, and F1-score.
4. **ROUGE-2:** The ROUGE-2 metric evaluates the bigram overlap, focusing on the accuracy of consecutive word pairs in the generated text compared to the reference. It calculates precision, recall, and F1-score based on bigram matches.
5. **METEOR:** Metric for Evaluation of Translation with Explicit Ordering (METEOR) (31) is a metric used for evaluating the quality of machine-generated translations by comparing them with human-generated reference translations. Unlike precision-focused metrics like BLEU, METEOR places more emphasis on recall and incorporates additional linguistic features such as stemming synonym matching, and paraphrase matching.

## Results

In this section, we present the results of evaluating our proposed model on the Indiana University Chest x-ray Dataset using various evaluation metrics, including BLEU, ROUGE, and METEOR. These metrics offer a thorough evaluation of the quality of the generated text in terms of recall and precision.

A comparison of our model's output to many cutting-edge methods for producing medical reports from chest x-ray pictures is shown in Table 1. All evaluation criteria, such as ROUGE-L, METEOR, and BLEU scores, show that our model performs better. We succeeded in capturing unigrams pertinent to the medical context with a BLEU-1 score of 0.675, which is noteworthy and outperforms previous models like Alqahtani et al. (35) and Singh et al. (36). In longer generated sequences, our BLEU-2, BLEU-3, and BLEU-4 scores, which are 0.585, 0.523, and 0.472, respectively, demonstrate great coherence and relevance. With a METEOR score of 0.382, our model outperforms many other models in producing linguistically diverse text, highlighting its efficacy in capturing semantic nuances. Additionally, a high degree of structural similarity between the generated reports and the reference texts is indicated by our ROUGE-L score of 0.698, indicating that our approach performs exceptionally well in preserving sentence-level organization. Overall, Table 1's results confirm that our model performs noticeably better than current approaches, highlighting its efficiency in automatically generating high-quality medical reports.

**Table 1:** Performance metrics of state-of-the-Art models across BLEU, METEOR, and ROUGE scores.

S. no	Works	BL-1	BL-2	BL-3	BL-4	MTR	RG-1	RG-2	RG-L
1.	Niksaz et al. (32) (ResNeXt + BioBert)	0.178	0.146	0.135	0.102	–			–
2.	Junior et al. (33)	0.377	0.239	0.168	0.124	0.322			0.300
3.	Yelure et al. (34) (Encoder-Decoder)	0.11	0.23	0.32	0.38	–			–
4.	Yelure et al. (34) (Encoder-Decoder with Attention)	0.11	0.32	0.46	0.56	–			–
5.	Alqahtani et al. (35)	0.479	0.363	0.261	0.173	0.188			0.354
6.	Singh et al. (36) (ResNet-101,CNN + Transformer)	0.311	0.196	0.131	0.091	0.136			0.264
7.	Shaikh et al. (37)	0.465	0.300	0.220	0.172	0.185			0.361
8.	Alfarghaly et al. (38) (CDGPT2)	0.387	0.245	0.166	0.111	0.164			0.289
9.	Akbar et al. (18)	0.558	0.463	0.311	0.097	–			0.448
10.	Raminedi et al. (39) (ViGPT2)	0.571	0.385	0.291	0.226	–			0.433
11.	Ours	0.675	0.585	0.523	0.472	0.382	0.72	0.55	0.698

The failure cases outlined in the (Table 2) highlights key limitations in the dataset and model, particularly the underrepresentation of rare or subtle conditions. For instance, the omission of calcified granulomas and degenerative changes underscores the challenge of detecting less common or subtle abnormalities that may not be adequately represented in the training data. Similarly, the model's failure to capture the nuanced description of acute bony findings points to difficulties in handling ambiguous or borderline cases. While the general findings were correctly identified, minor stylistic differences in phrasing reflect inconsistencies in reporting, though not affecting clinical accuracy. These failures emphasize the need for a more diverse dataset, with a better balance between common and rare conditions, to ensure the model can generalize effectively. Future improvements, such as data augmentation, synthetic data generation, and class balancing, will help address these gaps and enhance the model's ability to accurately detect a wider range of clinical findings, ultimately improving its robustness and applicability in real-world clinical settings.

**Table 2:** Limitations and failure cases.

S. no	Case	Sample report findings	Generated report findings	Issue
1.	Calcified granuloma	Large calcified granuloma within the medial right lung base	Granuloma not mentioned	Omission of rare finding (calcified granuloma)
2.	Degenerative changes	Mild degenerative changes at the lower thoracic spine	No mention of degenerative changes	Omission of subtle abnormality (degenerative changes)
3.	Bony abnormalities	Convincing acute bony findings	No acute bony abnormality	Ambiguous terminology; failed to capture nuanced description of bony findings
4.	General findings	Clear lungs, normal heart size, no pleural effusion or pneumothorax	Similar findings	Minor phrasing differences, no false analysis; just style variance

## Discussion

This work demonstrates promising results using the ChestX-Transcribe model, but several limitations related to both the dataset and the model itself must be considered. The Indiana University Chest x-ray (IU CXR) dataset, while valuable, may introduce selection bias due to its specific origins within the Indiana Network for Patient Care. This can affect the model's generalizability if certain patient demographics or less common medical conditions are underrepresented, limiting its applicability to a wider, more diverse population, including rural or international healthcare settings. Additionally, the dataset may not capture the full spectrum of conditions, such as rare findings like calcified granulomas or degenerative changes, which could result in omissions or misclassifications in model output. Regarding the model, DistilGPT was chosen for its balance between computational efficiency and coherence, but more advanced models such as GPT-4 or T5, fine-tuned for medical data, could provide more accurate and context-sensitive reports. However, these models come with higher computational costs, which could hinder scalability in real-world clinical applications, where timely report generation is crucial. Furthermore, since the model was trained on a single dataset, its performance on other datasets with differing characteristics—such as those containing rare conditions or subtle findings like bony abnormalities—remains uncertain. This underscores the need for further validation on diverse datasets to assess the model's robustness and generalizability. These limitations highlight the need for data diversity, improved model efficiency, and cross-dataset validation to enhance the model's practicality in real-world clinical settings.

## Conclusion

In this study, we aimed to develop a multimodal sequence-to-sequence transformer model for generating accurate medical reports from chest x-ray images, which addresses the critical need for automated systems in radiology. Our proposed model demonstrated superior performance across various evaluation metrics, achieving BLEU, ROUGE, and METEOR scores that outperformed several state-of-the-art models. The integration of a Swin Transformer for visual feature extraction and DistilGPT for text generation proved effective in producing coherent and contextually relevant medical narratives. The findings (Table 3) suggest that our method can markedly improve the efficiency of medical reporting, potentially assisting healthcare practitioners in delivering prompt and precise diagnoses. This automation could alleviate the workload on radiologists, allowing them to focus more on patient care.

**Table 3:** Example predictions of ChestX-transcribe.

Input image	Ground truth	Predicted
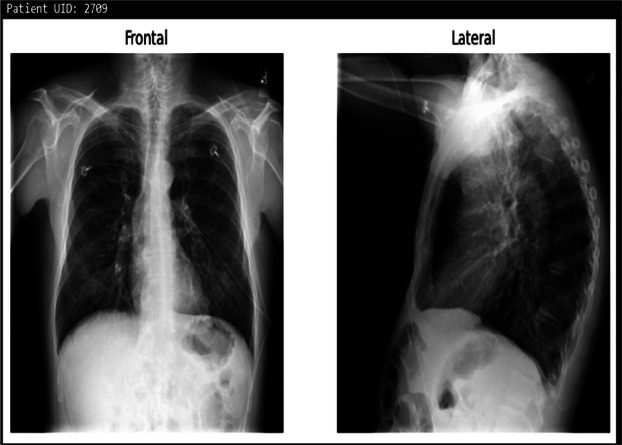	There is a subtle left medial base opacity. Cardiomediastinal silhouette is normal. Pulmonary vasculature and XXXX are normal. No pneumothorax or large pleural effusion. Osseous structures and soft tissues are normal.	XXXX are unchanged. No focal airspace consolidation. No pleural effusion or pneumothorax. Heart size and mediastinal contour are within normal limits.
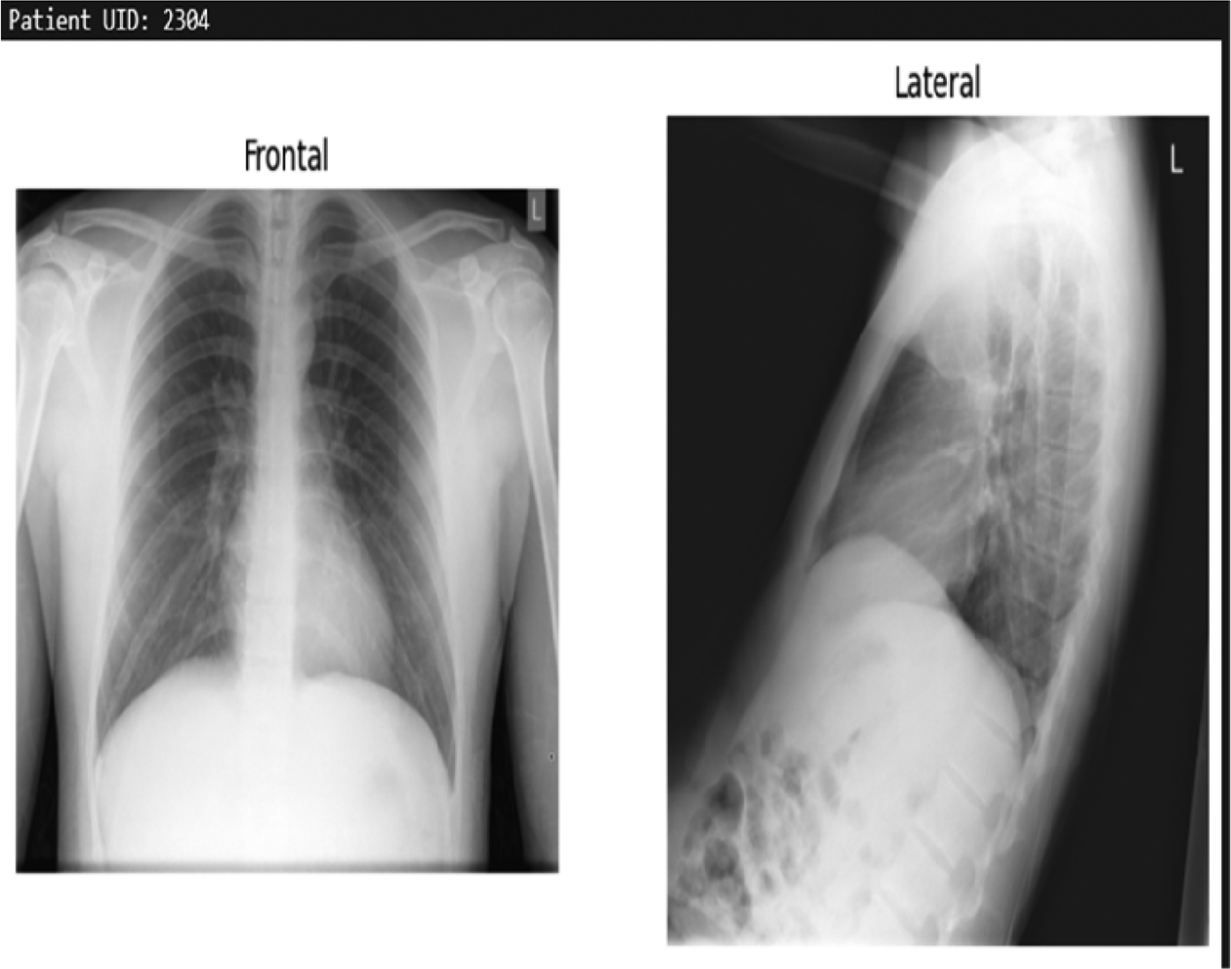	Cardiomediastinal silhouette and pulmonary vasculature are within normal limits. Lungs are clear. No pneumothorax or pleural effusion. No acute osseous findings.	C and lateral views of the chest. The cardiomediastinal silhouette is normal in size and contour. No focal consolidation, pneumothorax or large pleural effusion. No acute bony abnormality.
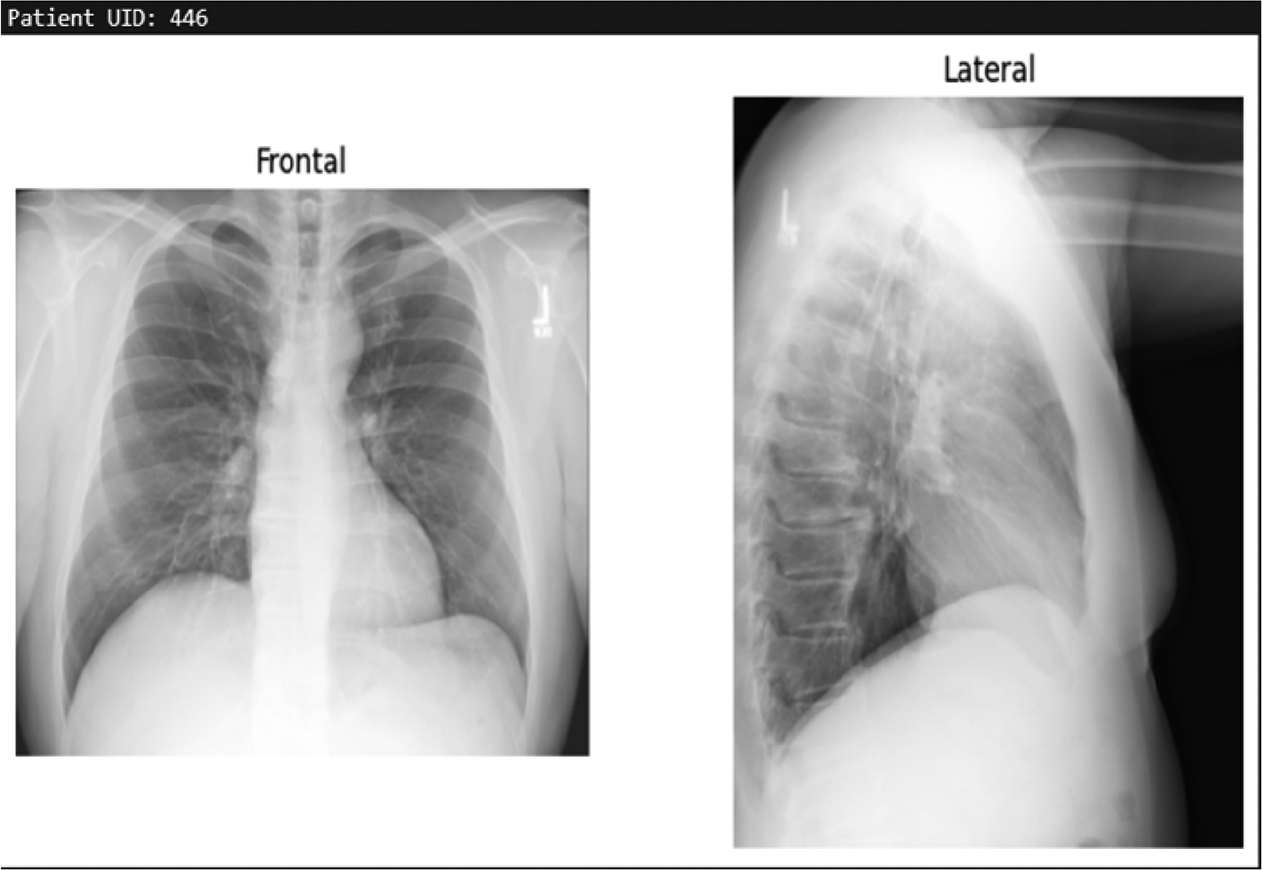	The cardiac contours are normal. The lungs are clear. Thoracic spondylosis.	Heart size and mediastinal contours. The lungs are clear. Thoracic spondylosis.
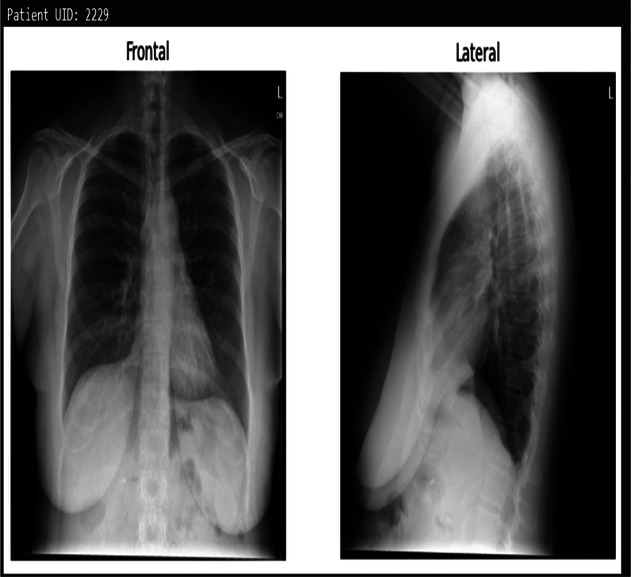	The heart size is normal. The mediastinal contour is within normal limits. The lungs are free of any focal infiltrates. There are no nodules or masses. No visible pneumothorax. No visible pleural fluid. The XXXX are grossly normal. There is no visible free intraperitoneal air under the diaphragm. Surgical clips are seen the right upper quadrant.	Heart size is normal. Lungs are clear.
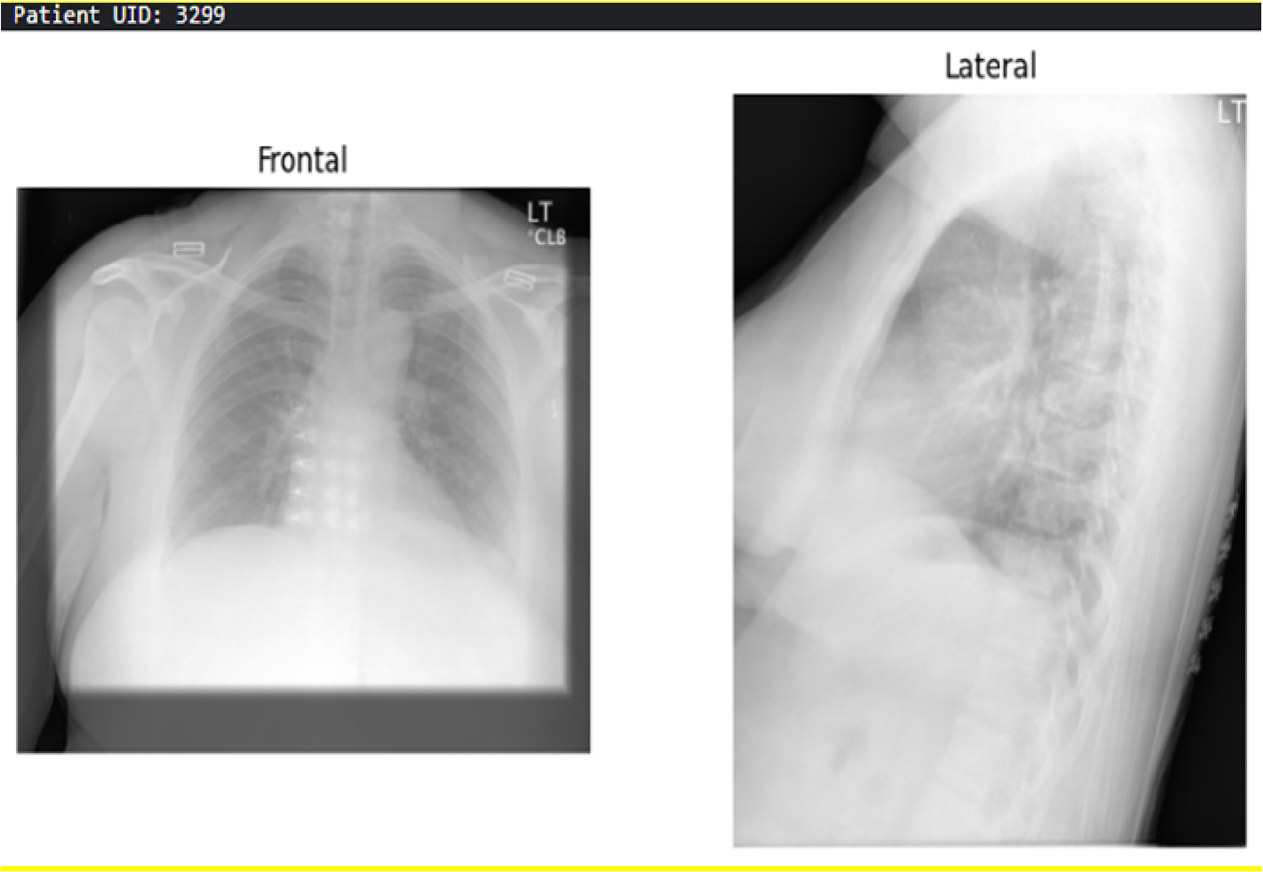	The cardiac silhouette and mediastinal contours are within normal limits. There are low lung volumes with bronchovascular crowding. Otherwise the lungs are clear. There is no pneumothorax. No large pleural effusion.	Low lung volumes. Heart size within normal limits. No pneumothorax or pleural effusion.
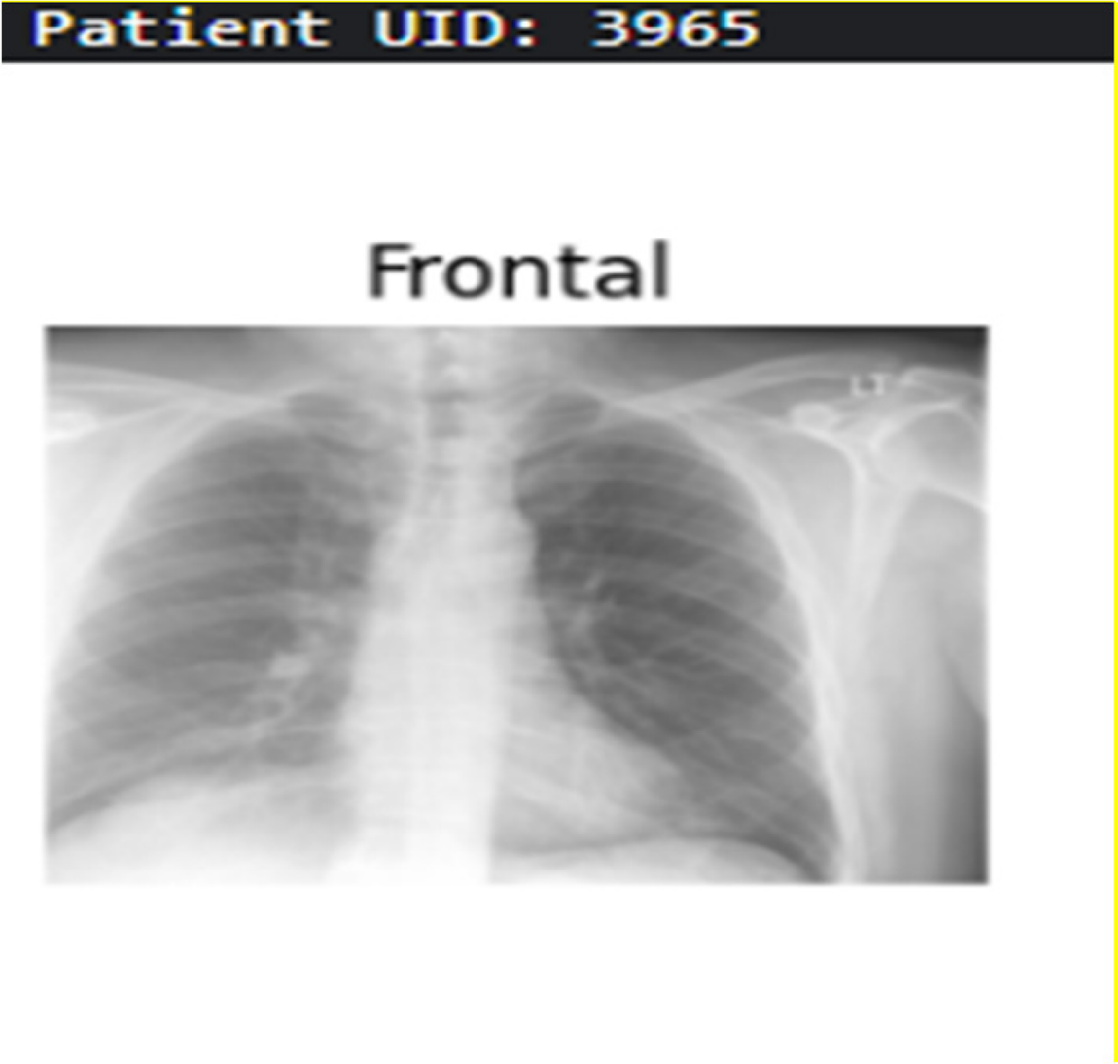	The heart and lungs have XXXX XXXX in the interval. Both lungs are clear and expanded. Heart and mediastinum normal.	XXXX opacities XXXX representing chronic lung disease. Lungs are clear. No pleural effusions or pneumothoraces. Heart and mediastinum of normal size and contour. Degenerative changes in the spine.
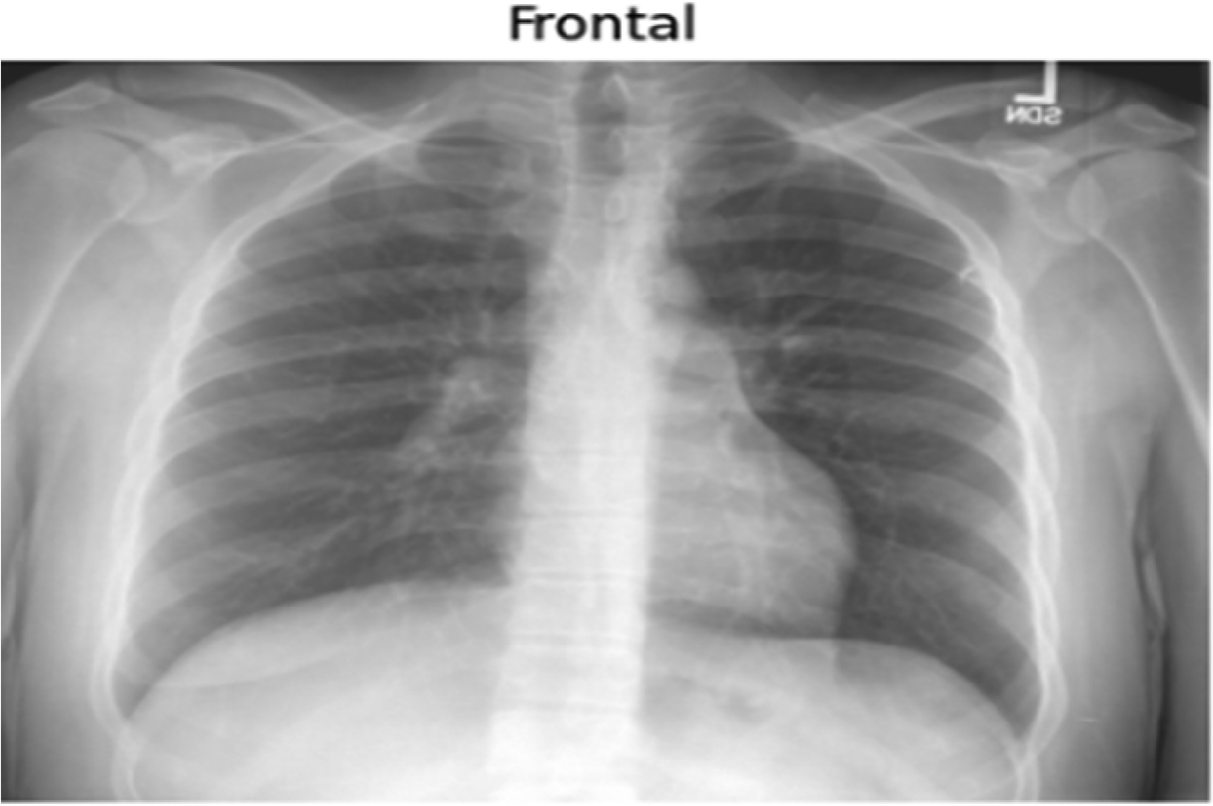	Right lower lobe XXXX calcified granuloma. Heart size within normal limits. No pleural effusions. No evidence of pneumothorax. Degenerative changes thoracic spine.	Right lower lobe XXXX calcified granuloma. Heart size and pulmonary vascularity within normal limits. No focal consolidation, pneumothorax or pleural effusion.

While our model performs well on the evaluated dataset, it is essential to recognize that the results may vary with different datasets or imaging modalities. Further exploration is needed to generalize the model's performance across various clinical contexts. As noted by Pan et al. (40), integrating large AI models into radiology workflows presents challenges, including data privacy concerns, ethical considerations, and compatibility with existing hospital infrastructure. Future work should focus on refining the model by incorporating larger and more diverse datasets to enhance its robustness. Additionally, exploring hybrid architectures or integrating attention mechanisms may yield further performance improvements.

To transition this model into real-world clinical systems, several steps should be considered. One key recommendation is to address data privacy concerns, ensuring the protection of patient information in compliance with healthcare regulations. Moreover, integration into existing hospital infrastructure, including compatibility with radiology workstations and electronic health records, would be essential for seamless deployment. Implementing real-time processing capabilities to enable timely report generation is another practical challenge. Ethical considerations surrounding the use of AI in healthcare, such as transparency, accountability, and bias mitigation, must also be discussed to ensure responsible adoption. Ultimately, this research lays the groundwork for the development of intelligent systems that not only improve the accuracy of medical reporting but also pave the way for innovative applications in automated healthcare diagnostics.

## Data Availability

The original contributions presented in the study are included in the article/Supplementary Material, further inquiries can be directed to the corresponding authors.
